# Chitosan-based nanodelivery systems applied to the development of novel triclabendazole formulations

**DOI:** 10.1371/journal.pone.0207625

**Published:** 2018-12-12

**Authors:** Daniel Real, Stefan Hoffmann, Darío Leonardi, Claudio Salomon, Francisco M. Goycoolea

**Affiliations:** 1 Instituto de Química de Rosario, Consejo Nacional de Investigaciones Científicas y Tecnológicas, Rosario, Argentina; 2 Departamento Farmacia, Facultad de Cs. Bioquímicas y Farmacéuticas, Universidad Nacional de Rosario, Rosario, Argentina; 3 Institute of Plant Biology and Biotechnology (IBBP), Westfälische Wilhelms-Universität Münster, Münster, Germany; 4 School of Food Science and Nutrition, University of Leeds, Leeds, United Kingdom; Catholic University of Korea, REPUBLIC OF KOREA

## Abstract

Triclabendazole is a poorly-water soluble (0.24 μg/mL) compound classified into the Class II/IV of the Biopharmaceutical Classification System. It is the drug of choice to treat fascioliasis, a neglected parasitic disease worldwide disseminated. Triclabendazole is registered as veterinary medicine and it is only available for human treatment as 250 mg tablets. Thus, the aim of this work was to develop novel drug delivery systems based on nanotechnology approaches. The chitosan-based nanocapsules and nanoemulsions of triclabendazole were fully characterized regarding their particle size distribution, polydispersity index and zeta potential, *in-vitro* release and stability in biological media. Cytotoxicity evaluation and cellular uptake studies using CaCo-2 cell line were also investigated. The results indicated an average hydrodynamic size around ~160 nm were found for unloaded nanoemulsions which were slightly increased up to ~190 nm for loaded one. In contrast, the average hydrodynamic size of the nanocapsules increased from ~160 nm up to ~400 nm when loaded with triclabendazole. The stability studies upon 30 days storage at 4, 25 and 37°C showed that average size of nanoemulsions was not modified with varying amounts of loaded TCBZ while an opposite result was seen in case of loaded nanocapsules. In addition, a slight reduction of zeta potential values over time was observed in both triclabendazole nanosystems. Release of TCBZ from nanoformulations over 6 h in simulated gastric fluid was 9 to 16-fold higher than with untreated TCBZ dispersion. In phosphate buffer saline solution there was no drug release for neither nanocapsules nor nanoemulsions. Cell viabilities studies indicated that at certain concentrations, drug encapsulation can lower its cytotoxic effects when compared to untreated drug. Confocal laser scanning microscopy study has shown that nanocapsules strongly interacted with Caco-2 cells *in vitro* which could increase the passage time of triclabendazole after oral administration. The results of this study constitute the first step towards the development of nanoformulations intended for the oral delivery of anti-parasitic drugs of enhanced bioavailability.

## Introduction

**Fig 1:**
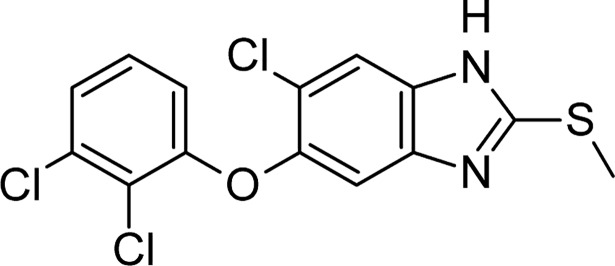
Chemical structure of triclabendazole.

Triclabendazole (TCBZ, 6-chloro-2-(methylthio)-5-(2,3-dichlorophenoxy)-1H-benzimidazole, Fig 1) is a highly lipophilic benzimidazole derivative classified into the class II/IV of the Biopharmaceutical Classification System (BCS) [1].

Its reported solubility in water is 0.24 μg/mL and its *log* P is 5.44. It is the drug of choice to treat fascioliasis, a neglected parasitic disease worldwide disseminated [2]. Fascioliasis is caused by *Fasciola hepatica*, a helminth parasite, and affect both domestic ruminants and humans [3]. A recent report describes that around 2.4 million people in the world are infected in different regions and more than 90 million are at risk [4]. TCBZ is active against both immature and adult parasites and its absorption is increased in presence of fatty foods. In general, few side effects have been reported including abdominal pain and headaches as well as biliary colic due to the passage of dead or dying parasites through the bile ducts. TCZB, registered as veterinary medicine, is only available for human treatment as 250 mg tablets manufactured by Novartis as Egaten®. It is worth noting that the recommended dose of TCBZ is 10 mg/kg in a single dose [5]. Based on this, in the case of pediatric treatments, the tablets should be divided in smaller pieces for dose adjustment and ease in swallowing. However, it could lead to adverse effects due to a potential incorrect dosage. In addition, ingestion of tablet fragments may be a serious drawback in terms of flavour and palatability, particularly for pediatric patients. Therefore, other appropriate dosage forms to provide lower doses in the case of paediatric patients, are highly required [6].

It is well-known that one of the more critical challenges in the pharmaceutical industry is the design of novel drug delivery systems with improved biopharmaceutical properties [7,8]. Lipophilic compounds, such as TCBZ, may easily interact with the target receptor leading to the desired biological activity. However, the lack of water solubility of such lipophilic molecules is the rate-limiting step for drug absorption and/or membrane permeability [9,10]. Several strategies have been used to improve the aqueous solubility and further bioavailability of drugs including the development of solid dispersions, complexation with cyclodextrins and polymeric microencapsulation [11,12,13,14,15]. Lately, reduction of the particle size from micro- to nanometer range has been recognized as a promising approach to improve the apparent saturation solubility, dissolution rate and oral bioavailability of hydrophobic molecules (BCS Class II/IV drugs) [16,17]. When compared with microparticulate delivery devices, particles of small size (<1 **μ**m) are likely to accumulate in the site of action, increase the concentration at the target site and hence, improve the therapeutic efficacy. Moreover, nanoparticles are capable to co-associate/co-encapsulate more than one drug of different type and protect them from their chemical or enzymatic degradation [18,19].

The use of nanoparticles to treat parasite diseases has proven to be effective [20]. Particularly, the development of nanomedicines has been widely investigated as an attractive alternative to improve the *in vivo* performance of several hydrophobic antiparasite drugs. Thus, Rial *et al*. reported the formulation an *in vivo* evaluation of benznidazole nanocrystals for the treatment of Chagas disease [21]. Particularly, they observed a remarkable effect of low doses of nanoformulated benznidazole on immunosuppressed infected mice, indicating the potential importance of nanoformulated BNZ in experimental therapy. Paredes *et al*. evaluated the dissolution and bioavailability performance of albendazole nanocrystals and their results confirmed improved pharmacokinetic parameters and a significant *in vivo* efficacy observed for the novel nanoformulated albendazole in comparison with the conventional treatment [22]. In addition, praziquantel was nanoformulated into a montmorillonite clay carrier by El-Feky *et al*. [23]. It was found that this novel nanosystem increased the drug dissolution rate and, therefore, the oral absorption and bioavailability of praziquantel compared to the untreated drug. On the other hand, Mourão *et al*. reported the formulation of praziquantel phosphatidylcholine-containing liposomes. The *in vivo* studies demonstrated that liposomes with PZQ decreased the amounts of eggs and parasites improving, as a consequence, the antischistosomal efficacy of the drug [24]. However, to the best of our knowledge, the development and characterization of nanodelivery systems of TCBZ has not been addressed.

Nanoemuslions (NEs) are biphasic colloidal dispersions of two immiscible liquids, namely either water-in-oil or oil-in-water, both stabilized by an amphiphilic surfactant. On the other hand, nanocapsules (NCs), in which the drug payload is confined in a reservoir or within a cavity, are surrounded by a coating. Recent studies show that biopolymers, such as chitosan or cellulose and modifications of them, are ideal candidates for developing improved coated nanocapsule formulations with increased efficiency in the delivery of different drugs, mainly of the hydrophobic type [25]. Both NEs and NCs have average hydrodynamic diameters in the sub-micron range, typically ~100 to ~500 nm. Therefore, the aim of the present study was to investigate whether the application of nanotechnology would be a convenient approach to formulate chitosan-based nanocapsules (NCs) and nanoemulsions (NEs) loaded with TCBZ. The formulated TCBZ nanosystems were fully characterized regarding their particle size distribution, polydispersity index and zeta potential. *In vitro* release studies and stability in biological media were also performed. In addition, cytotoxicity evaluation and cellular uptake studies were carried out using CaCo-2 cell line.

## Materials and methods

### Materials

TCBZ was purchased from Chemo (Buenos Aires, Argentina). Chitosan 70/5 (batch 212-170614-01) was purchased from HMC+ GmbH (Halle-Saale, Germany). Miglyol® 812 was kindly provided by Peter Cremer Oleo (USA). The surfactant lecithin (Epikuron® 145v, a phosphatidylcholine enriched fraction of soybean lecithin) was kindly donated by Cargill (Spain). Trehalose dehydrate was purchased from Sigma-Aldrich (Spain). All water used in the study was ultrapure MilliQ water with a resistivity of 18.2 MΩ at 25°C and passed through a filter with a pore size of 0.22 μm. All other reagents used were of analytical grade if not specified otherwise.

## Methods

### Formulation of nanocapsules and nanoemulsions

NCs and NEs were prepared according with the protocol first described by Calvo *et al*. [26] with slight modifications. The overall principle of this method is known as solvent displacement or spontaneous emulsification [27,28,29]. Briefly, an organic phase was formed by dissolving 40 mg of lecithin in 0.5 mL of ethanol, followed by the addition of 125 μL of Mygliol® 812 (a neutral oil formed by esters of caprylic and capric fatty acids and glycerol) and adding ethanol up to 10 mL. This 10 mL volume of organic phase were immediately poured over 20 mL of the aqueous phase composed of a chitosan solution (0.5 mg/mL in water, stoichiometrically dissolved with 1 M HCl with 5% excess) in case of NCs, or in 20 mL of water in case of NEs. Both NCs and NEs were formed spontaneously due to the organic solvents diffusion. Finally, the ethanol and part of the water were evaporated at 40°C under vacuum on a R-210 Rotavapor (Büchi Labortechnik GmbH, Essen, Germany) and the volume of the formulations was reduced to 10 mL.

### Physicochemical characterization

The systems were characterized regarding their size, size polydispersity and zeta potential. Particle size and polydispersion index were determined by dynamic light scattering with non-invasive back scattering (DLS-NIBS) with a measurement angle of 173°. The autocorrelation functions were fitted with the default Non-Negative Least Squares (NNLS) fit to calculate the intensity size distribution plots and evaluate the Z-average diameter. The zeta potential was measured by mixed laser Doppler velocimetry and phase analysis light scattering (M3-PALS). A Malvern Zetasizer NanoZS (Malvern Instruments, Malvern, UK) fitted with a red laser (λ = 632.8 nm) was used for both methods. Samples were diluted 1:100 before the evaluations which were carried out in triplicate. The Zeta Sizer Software (v 7.12) was used to acquire and evaluate the size and zeta potential data.

### Association and loading efficiency

Following an identical procedure already described, the association efficiency of the formulated TCBZ NCs and NEs aqueous solutions was calculated after the separation of drug insoluble crystals (non-encapsulated) by ultracentrifugation (Mikro 220 R, Hettich GmbH & Co. KG, Tuttlingen, Germany) at 16000 rpm for 10 minutes at 15°C. After this procedure, the TCBZ nanoformulations formed a creamy layer on top of the aqueous phase, while the suspended crystals precipitated forming a pellet at the bottom of the vial. The associated TCBZ was determined by resuspending the creamy layer with distilled water up to 1 ml and then 50 μl were taken and mixed with 450 μl of absolute ethanol. TCBZ content was determined by UV spectrophotometry (Jasco, Gross-Umstadt, Germany) at λ = 305 nm against a standard curve produced with drug stock solution. The association efficiency was calculated as the difference between the total amount of TCBZ incorporated in the formulation and the amount present in the filtrate and the pellet:

$$Association\;efficiency\;(\%)=\frac{{Mass\;drug}_{associated}}{{Mass\;drug}_{theoretical}}*100$$

The loading efficiency expresses how much mass of the nanosystem consists of drug as opposed to molecules that make the structure of the nanosystem and was calculated using the following equation:

$$Loading\;efficiency\;(\%)=\frac{{Mass\;drug}_{associated}}{{Mass}_{Nanosystem}}*100$$

### Stability of TCBZ nanoformulations during storage

Aliquots of every system were kept in sealed tubes at different temperatures (4, 25 and 37°C). The particle size of the nanoformulations was monitored every week over a period of 1 month and zeta potential values were controlled every 15 days for a period of 1 month. Macroscopic aspects (presence of aggregated, cream formation, flocculation, coalescence, changes in colour, etc.) were also assessed throughout the period of study.

### Equilibrium solubility at different pHs

TCBZ saturated solubility at different pH values was calculated by adding an excess amount of TCBZ (100 mg) to 10 mL of buffer (pH 1–8). The hermetically sealed flasks were shaken (180 rpm) for 72 h in a water bath at 25±0.5°C. After equilibrium was reached (4 h), the suspensions were filtered through cellulose nitrate membranes (0.45 μm pore size), and the concentration of TCBZ in solution was determined by UV spectrophotometry at 305 nm (Boeco S26 spectrometer, Hamburg, Germany). Each experiment was carried out in triplicate.

### *In vitro* triclabendazole release assay

A sample of each formulation (800 μL) was transferred to a standarized dialysis tube with an area of 2 cm^2^ (Pure-a-lyser Maxi 0.1–3.0 mL, Mw cut off 6kDa, Sigma-Aldrich GmbH, Steinheim, Germany) and placed in a glass beaker containing 79.2 mL of the medium previously equilibrated at 37°C in an incubator. All the experiments were performed using the same type of dialysis tubes. The TCBZ dose in each tube was 0.2, 5 and 10 mg for systems loaded with 0.25, 6.25 and 12.5 mg/mL, respectively. A 12.5 mg/mL TCBZ dispersion were used as reference.

### Stability in biological media

The stability of NCs and NEs during incubation in simulated gastric fluid (SGF, pH 1.2) and phosphate buffered saline (PBS, pH 7.4) was studied based on the behaviour of the particle size distribution at different time intervals (up to 6 h) at 37°C.

### Cytotoxicity assay

The cytotoxicity of the nanoformulations and components was evaluated in Caco-2 (RRID:CVCL_0025) colon adenocarcinoma cells by means of the MTT assay. Briefly, 100 μL of cell suspension was transferred to each well of a 96-well tissue culture plate (~10^4^ cells per well or ~10^5^ cells/mL) and cultured for 24 h. The cells were washed twice with supplement-free MEM before the sample was added and the cells were incubated for 3 h. The samples were removed and replaced with 100 μL supplement-free MEM. A MTT solution in PBS with a concentration of 5 mg/mL of thiazolyl blue tetrazolium bromide was prepared and added (125 μL) to each well. After 4h, the solution was removed again and the dye crystals dissolved in 100 μL DMSO. After orbital shaking at 300 rpm for 15 min, the absorbance was measured at λ = 570 nm in a microplate reader (Safire, Tecan AG, Salzburg, Austria). Relative viability values were calculated by dividing individual viabilities by mean of the medium control. Triton X-100 (4%) in PBS was used as a positive control [30].

### Cellular uptake studies

Confocal laser scanning microscopy was used to visualize the localization of NCs fluorescently labelled using 0.079 μM DiD (1,1´-dioctadecyl-3,3,3´,3´-tetramethylindodicarbocyanine, 4-chlorobenzenesulfonate salt), a lipophilic dye that showed complete association with NCs, in the presence of CaCo-2 cells. A sample of 10,000 cells were seeded and cultured for 2 days on glass slides (Ibidi μ-Dish, Martinsried, Germany). After removing the treatments cells were stained using CellMask™ Orange Plasma Membrane Stain (Thermo Fisher, Waltham, USA) following the protocols provided by the manufacturer.

### Statistical analysis

GraphPad Prism 7.0 software (GraphPad Software, USA) was used. Two way ANOVA test and Dunnett’s post-test were used for the multiple statistical comparisons of the release data obtained from the different systems. MTT data were analysed using a Two way ANOVA test and Tukey post-test for the multiple statistical comparisons. A value of P < 0.05 was considered statistically significant.

## Results and discussion

### Physicochemical characterization

**Fig 2:**
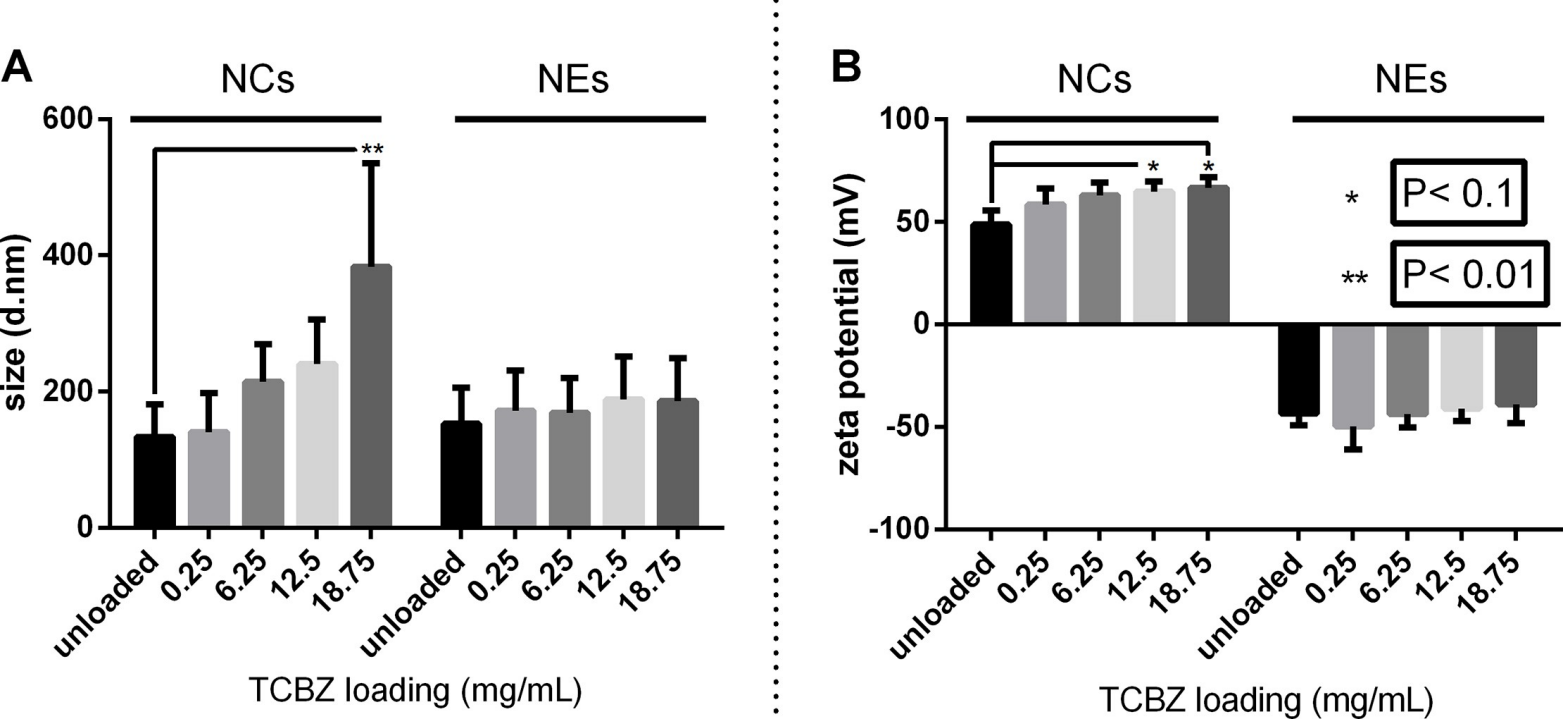
Physicochemical characterization of unloaded and TCBZ-loaded nanosystems. Z-average size (A) and Zeta potential (B) of pristine and triclabendazole (TCBZ)-loaded nanocapsules (NCs) and nanoemulsions (NEs) at varying drug loadings (data represent average values ± PDI width (nm)/zeta deviation (mV)).

The physicochemical analysis of NEs and NCs without and with increasing amounts of TCBZ is shown in Fig 2 Average hydrodynamic sizes around ~160 nm were found for unloaded NCs and NEs which were slightly increased up to ~190 nm for NEs loaded with up to 18.75 mg/mL of TCBZ. These results are in good agreement with previously reported characterization data for these systems loaded with different lipophilic bioactive phytochemicals [31,32,33]. Interestingly, the size of NCs increased significantly, the greater the drug loading was, showing a consistent trend. A possible interpretation to this would stem on the interaction of the drug with the lecithin-chitosan interface leading to the partial detachment of chitosan from its anchoring points, thus leading to an overall swelling of the polymer shell. Although the precise nature of the interactions of chitosan and lecithin at the interface of the nanocapsules is not fully understood, electrostatic and hydrophobic forces are known to be at play [34]. TCBZ can be envisaged as partioned preferentially in the oil core of the NCs, however, we cannot rule out that a fraction of it localizes also at the interface and disrupts the lecithin-chitosan interactions. Chitosan and lecithin interact predominantly via elecrostatic self-assembly as extensilvey documented in previous works [35,36].

This is further explained below when accounting for the effect of storage temperature on the stabililty of these systems. As can be observed in Fig 2A, the average hydrodynamic diameter of the NCs increased from its original magnitude (~160 nm) up to ~400 nm when loaded with 18.75 mg/mL of TCBZ.

On the other hand, polydispersity index (PDI) was found to be invariably below 0.2 for all formulations. In this regard, it is worth noting that those formulations that had a large increase in size when loaded, as compared to unloaded formulations, did not show an increase in their PDI, thus suggesting that the high drug loading capacity shown by these systems did not compromise their stability.

Zeta potential (ZP) measurements (Fig 2B) have shown, as expected, highly positive values for NCs and equally highly negative values for NEs that match previously reported values [31,32,33]. Higher drug loading increased ZP slightly but consistently for NCs as the drug loading increased. In the case of NEs, this effect was not observed. As can be observed, ZP was increased up to +60 mV when drug loading was 18.75 mg/mL of TCBZ. This increase in ZP with drug loading further supports our previous hypothesis that TCBZ is able to localize in the lecithin/oil interface and decrease the lecithin-chitosan interaction, thus leading to a swelling of the polymer shell, which could increase the influence of the positively charged amine groups of CS on the ZP.

**Fig 3:**
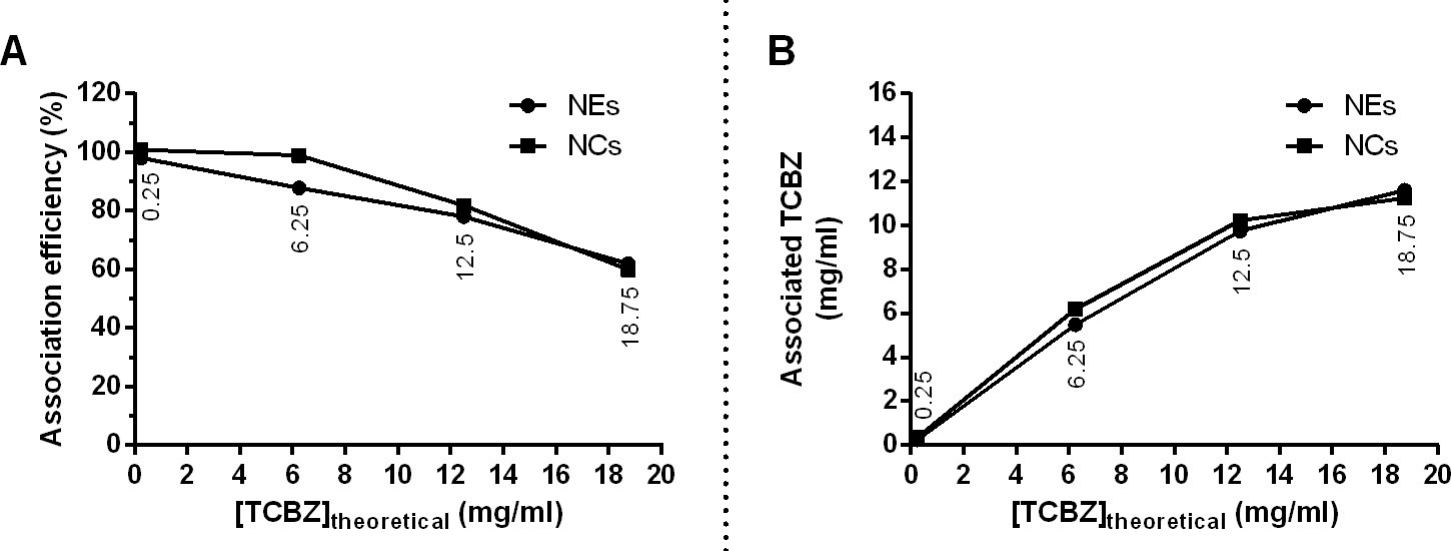
Association of triclabendazole (TCBZ) with nanosystems. Variation of association efficiency (A), and associated TCBZ (B) with the theoretically loaded TCBZ concentration in nanocapsules (NCs) and nanoemulsions (NEs).

The drug loading and total drug content of nanoformulations are shown in Fig 3. The association efficiency decreased with increasing concentration of TCBZ in the organic phase during preparation of the nanosystems (Fig 3A). On the other hand, the amount of loaded drug was essentially identical when the drug concentration in the organic phase was 12.5 mg/mL between NCs and NEs, with 10.0 and 9.7 mg/mL, respectively. Drug concentrations greater than 12.5 mg/mL in the organic phase, reduced the percentage of association efficiency (Fig 3A) and slightly improved the amount of drug (mg/mL) included in the nanoformulations (Fig 3B).

In Table 1 are shown the summary of the characteristics of the loaded and unloaded NCs and NEs, including the association efficiency and loading capacity. The association efficiency values determined correspond well with those measured in previous studies using chitosan-tripolyphosphate nanoparticles that lied in the range of 79.57 ± 0.96% and 13.38 ± 0.44% for the loading capacity [37].

**Table 1:** Summary of physical characteristics of pristine and triclabendazole (TCBZ)-loaded nanoemulsions (NE) and nanocapsules (NC) at varying drug loadings.

[TCBZ]_theoretical_ (mg/mL)	System	Z-av. diameter ± PDI width (nm)	Zeta potential ± zeta deviation (mV)	Association efficiency (%)	Loading efficiency (%)
**unloaded**	**NE**	153 ± 53	-43 ± 6	-	-
**0.25**		172 ± 58	-50 ± 11	98.00	1.51
**6.25**		169 ± 52	-44 ± 6	87.84	25.69
**12.50**		189 ± 63	-41 ± 6	78.06	38.06
**18.75**		186 ± 64	-39 ± 9	61.93	42.24
**unloaded**	**NC**	134 ± 48	49 ± 7	-	-
**0.25**		141 ± 57	59 ± 8	100.80	1.47
**6.25**		215 ± 56	63 ± 6	98.99	26.82
**12.5**		240 ± 66	65 ± 5	81.81	37.73
**18.75**		383 ± 153	67 ± 5	59.95	39.98

TCBZ association efficiency and loading efficiency (n = 3).

### Storage stability

**Fig 4:**
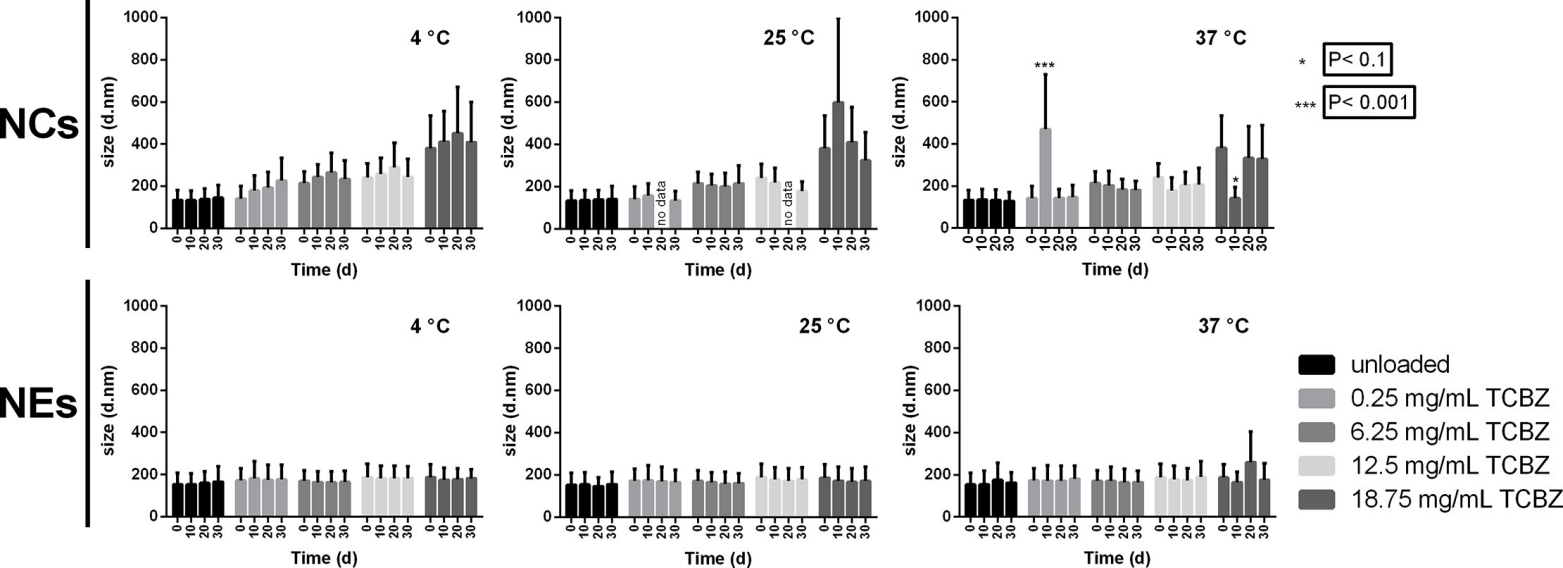
Size stability of nanosystems stored at different temperatures over 30 days. Variation of the z-average size of nanocapsules (NCs) and nanoemulsions (NEs) with increasing amounts of triclabedazole (TCBZ)—loading during storage at 4, 25 and 37°C for 0, 10, 20 and 30 days. Error bars represent PDI width in nm.

The stability of blank and TBCZ-loaded NCs and NEs was evaluated regarding the evolution of the size upon long-term storage at varying temperature, as shown in Fig 4. Of note, NE sizes were stable over time, regardless the storage temperature, with virtually no change in the average diameter values between the formulations with varying levels of loaded TCBZ. The analysis of the NCs, at 4°C, indicated a systematic increase in average size with storage time and this effect became more pronounced with higher TCBZ loading. On the other hand, at 25 and 37°C the size remained almost unaltered, except for the formulations with the highest TCBZ load. Particularly, the evolution of the size observed only on TCBZ-loaded NCs stored at 4°C, but not at higher temperatures, seem to point to the possible existence of an hydrophobic mechanism at play. Given the highly lipophilic nature of TCBZ, we reason that its presence at the NCs surface might result on the formation of a thicker shell driven by hydrophobic hydration of TCBZ. In any case, following the ANOVA results, no significantly differences were observed during 30 days, at 4, 25 and 37°C.

**Fig 5:**
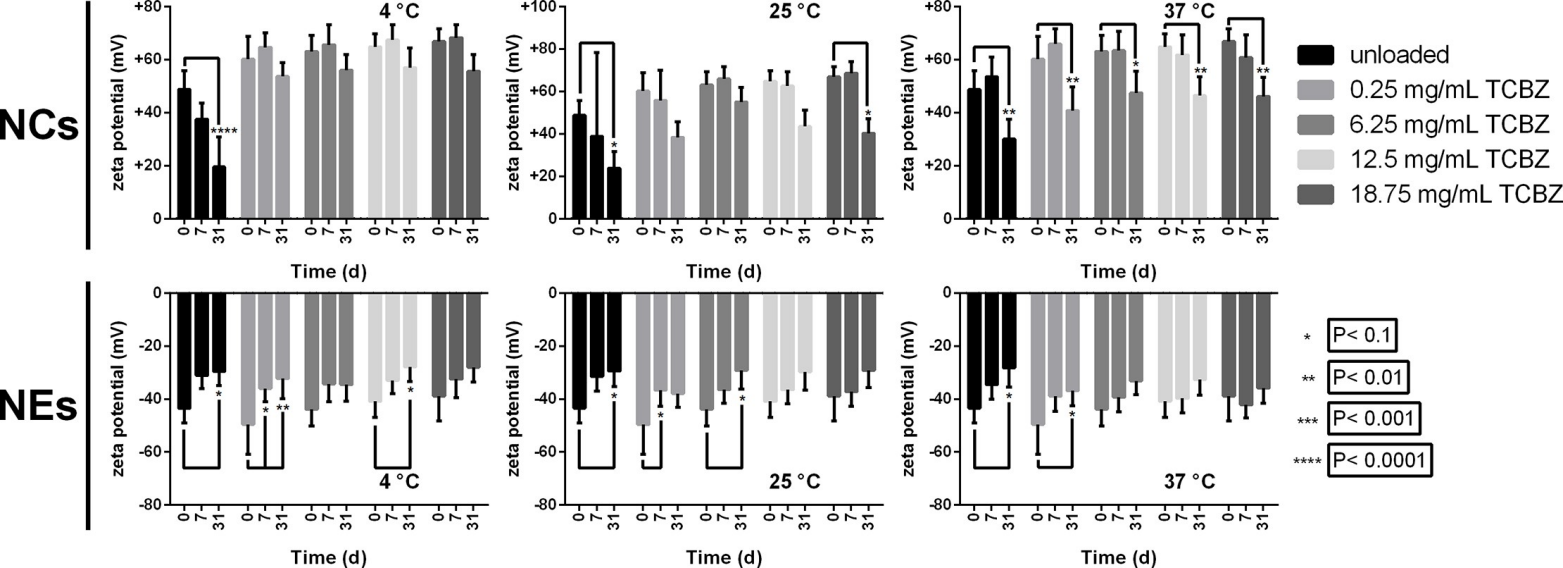
Zeta potential stability of nanosystems stored at different temperatures over 30 days. Variation of the zeta potential of nanocapsules (NCs) and nanoemulsions (NEs) with increasing amounts of triclabedazole (TCBZ)—loading during storage at 4, 25 and 37°C for 0, 10, 20 and 30 days. Error bars represent zeta deviation in mV.

As can be seen in Fig 5 for the corresponding variation in ZP, a significant reduction of the values of this parameter over time (31 days) was observed in all formulations of NCs and NEs. This behaviour was most pronounced in blank NCs than in TCBZ-loaded ones. While in NCs the ZP was unaltered until day 7, NEs showed to be less stable and their ZP decreased sooner. The storage temperature did not seem to influence the overall variation of the ZP for the various formulations. The decrease in ZP in TCBZ-loaded formulations is consistent with the proposal of the detachment of chitosan from the NC’s surface, as suggested above.

### Stability in biological media

**Fig 6:**
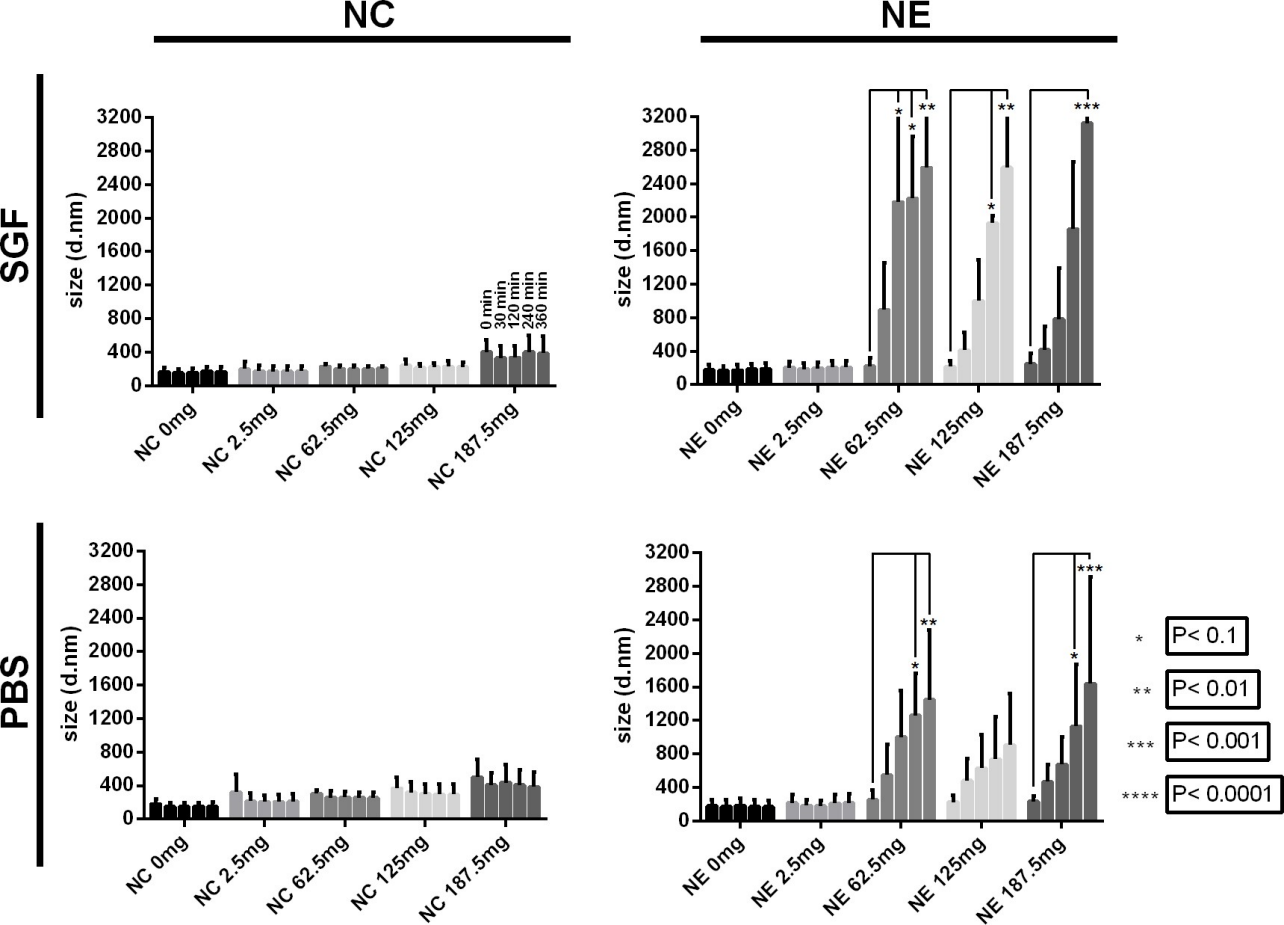
Size stability of nanosystems in different media over 6 hours. Variation of the z-average size of nanocapsules (NCs) and nanoemulsions (NEs) with increasing amounts of TCBZ-loading during incubation in simulated gastric fluid (SGF) and phosphate buffer saline (PBS) at 37°C during 360 min. Data represent average values, error bars represent PDI width in nm.

The stability of NCs and NEs during incubation in simulated gastric fluid (SGF, pH 1.2) and phosphate buffered saline (PBS, pH 7.4) was studied based on the behaviour of the particle size distribution at different time intervals (up to 6 h) at 37°C (Fig 6). As observed, NCs conserve their size over time in both SGF and PBS independently of the initial TCBZ loading. On the other hand, a systematic increase in average size was found in NEs. According to the ANOVA results, significantly differences were observed for NEs samples loaded with 62.5 and 187.5 mg/mL, respectively. Hence, the stability of the NCs is not only governed by an electrostatic repulsion but also due to repulsive hydration forces as previously shown by Santander-Ortega *et al*., which, in our experiments, seemed to be unaffected by TCBZ loading [38].

### Equilibrium solubility at different pHs and release of TCBZ in biorelevant media

**Fig 7:**
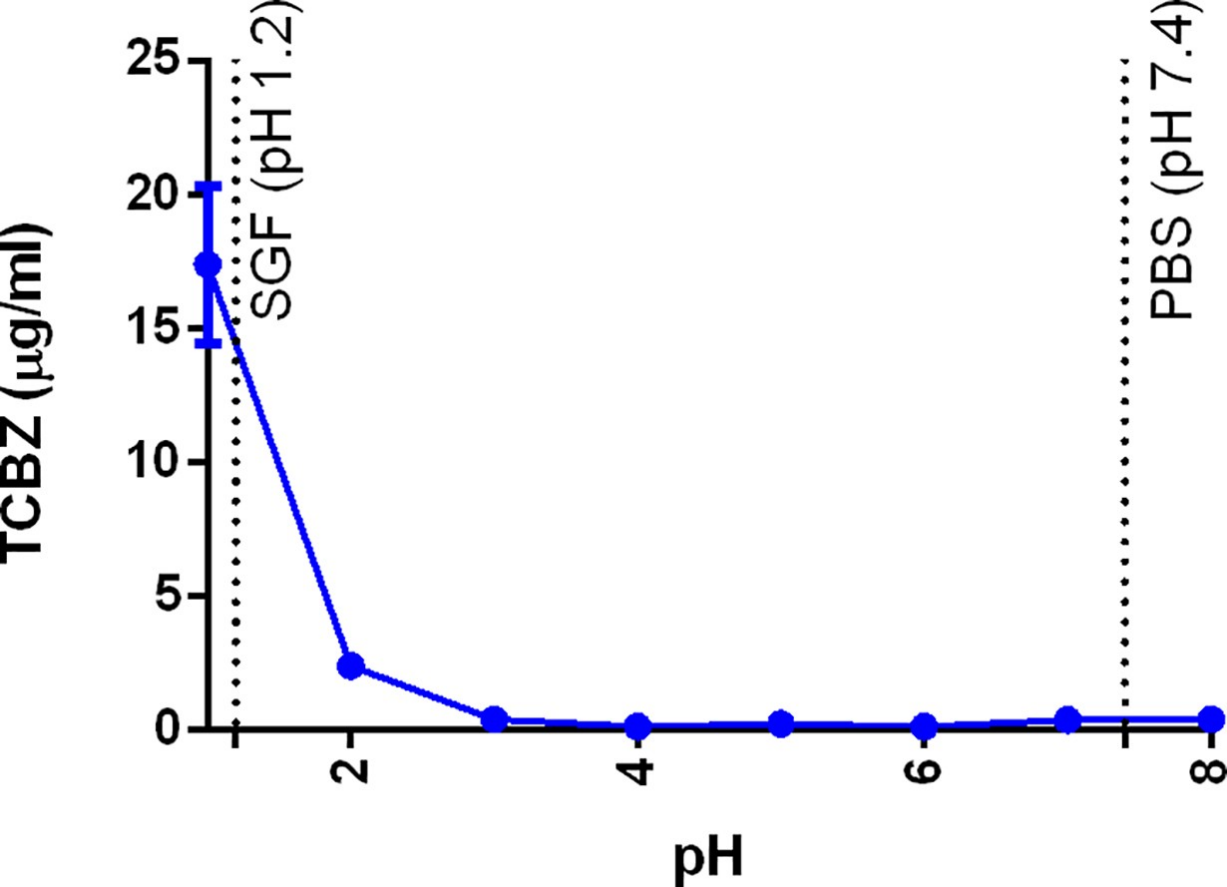
TCBZ solubility at different pHs.

As other related lipophilic benzimidazole derivatives, TCBZ may be ionizable at low pH values [14], and such ionization may greatly influence both further solubilisation and its release profile from nanodelivery systems. Thus, in this study, the solubility and release of TCBZ values was examined as a function of the pH (Fig 7).

**Fig 8:**
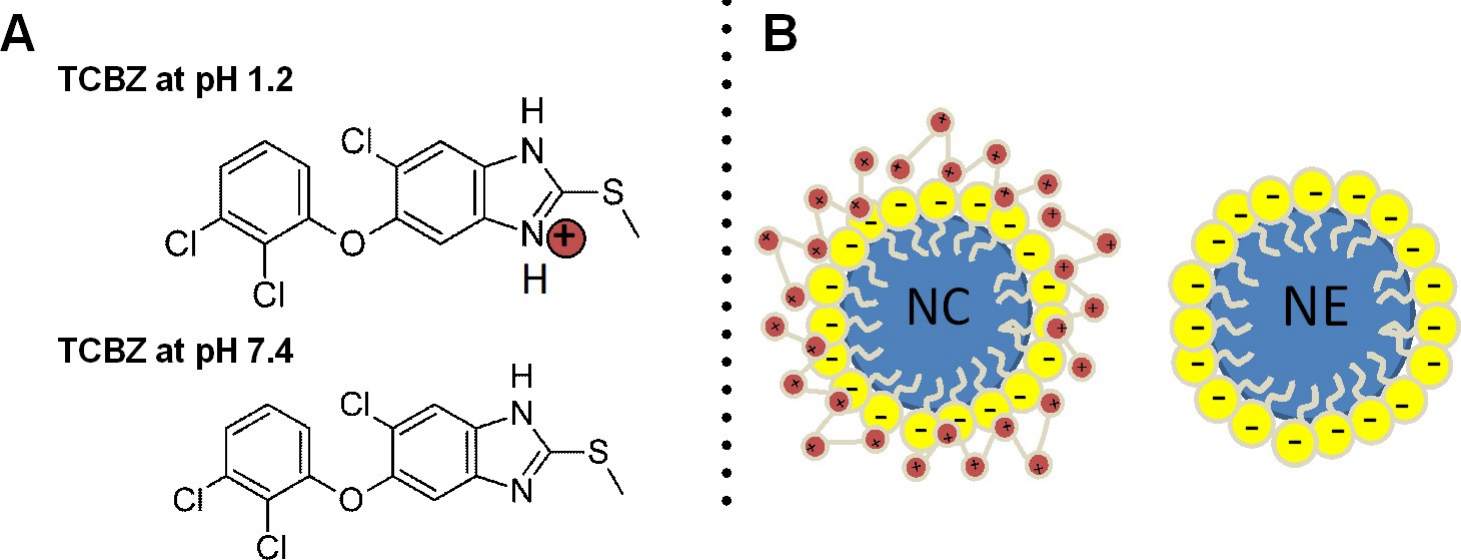
Graphical representation of TCBZ structure in different mediums (A), and structural scheme of NCs and NEs (B).

In Fig 7, the pH corresponding with simulated gastric fluid and phosphate buffered saline are indicated by dashed lines. In correspondence with chemically-related drugs (e.g. albendazole), TCBZ exhibited far greater solubility at pH 1 (17.37 μg/mL), than at pH 7 (0.35 μg/mL), probably due to the protonation of the imidazole ring at the lower pH value (Fig 8A) [39].

**Fig 9:**
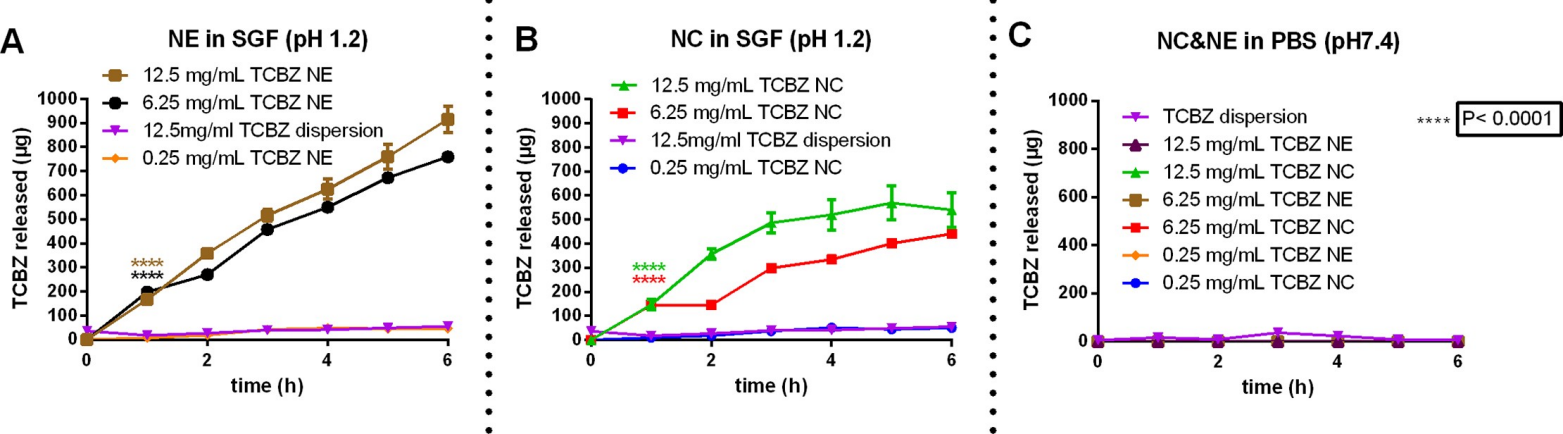
TCBZ release from nanosystems in different buffers.

Once the influence of pH over the TCBZ solubility was characterized, the release of TCBZ from NC and NE in different pH values relevant to the oral physiological context was analysed (Fig 9). Inspection of Fig 9A reveals that in simulated gastric fluid (SGF, pH 1.2) after 6 h incubation, untreated TCBZ, exhibited a total “release” of soluble drug of 52 μg. This value increased to more than 9- and 16-fold in NC and NE, respectively, loaded with 12.5 mg/mL. Of note, TCBZ was released from NEs under an almost linear behaviour over 6 h, with total release, as expected, depending on the initial amount of TCBZ loaded, reaching up to ~23 μg in formulations loaded with 0.25 mg/mL, and ~750 to ~910 μg for formulations loaded with 6.25 or 12.5 mg/mL, respectively.

Fig 9 also illustrates the TCBZ release profiles for NE (Fig 9A) and NCs (Fig 9B) in SGF (pH 1.2) and both NCs and NEs in PBS (pH 7.4) (Fig 9C) at different drug loadings and in comparison, with dispersed TCBZ. Notice in Fig 9B, that the released amounts of TCBZ were 25 μg in formulations loaded with 0.25 mg/mL and of ~400 to ~500 μg for formulations loaded with 6.25 or 12.5 mg/mL, respectively. These formulations with high TCBZ load, showed a negative deviation from linearity. The ionization of the molecule at pH 1.2 (Fig 8A) probably affects this release behaviour and, in case of NC, the electric impediment due the presence of the positive charge of chitosan slows down the release of the drug from the nanosystem (Fig 8B), leading to the observed non-linear release profile. In phosphate buffer saline (PBS, pH 7.4), we found that no TCBZ was released for neither NCs nor NEs, regardless of initial TCBZ loading, thus indicating that the *in vitro* release of TCBZ seems to be governed directly by the solubility of the drug at the given pH of each of the simulated media (Fig 9C). The results of the *in vitro* TCBZ release profile n simulated gastric conditions, showing the strong influence of the pH on the behaviour of the NE and NC formulations, allows to anticipate the behaviour of these formulations during oral delivery. The results also confirm that the association of TCBZ in the oil-core formulations results in a substantial increase on the overall drug solubility in gastric conditions.

### Cytotoxicity of TCBZ nanoformulations

**Fig 10:**
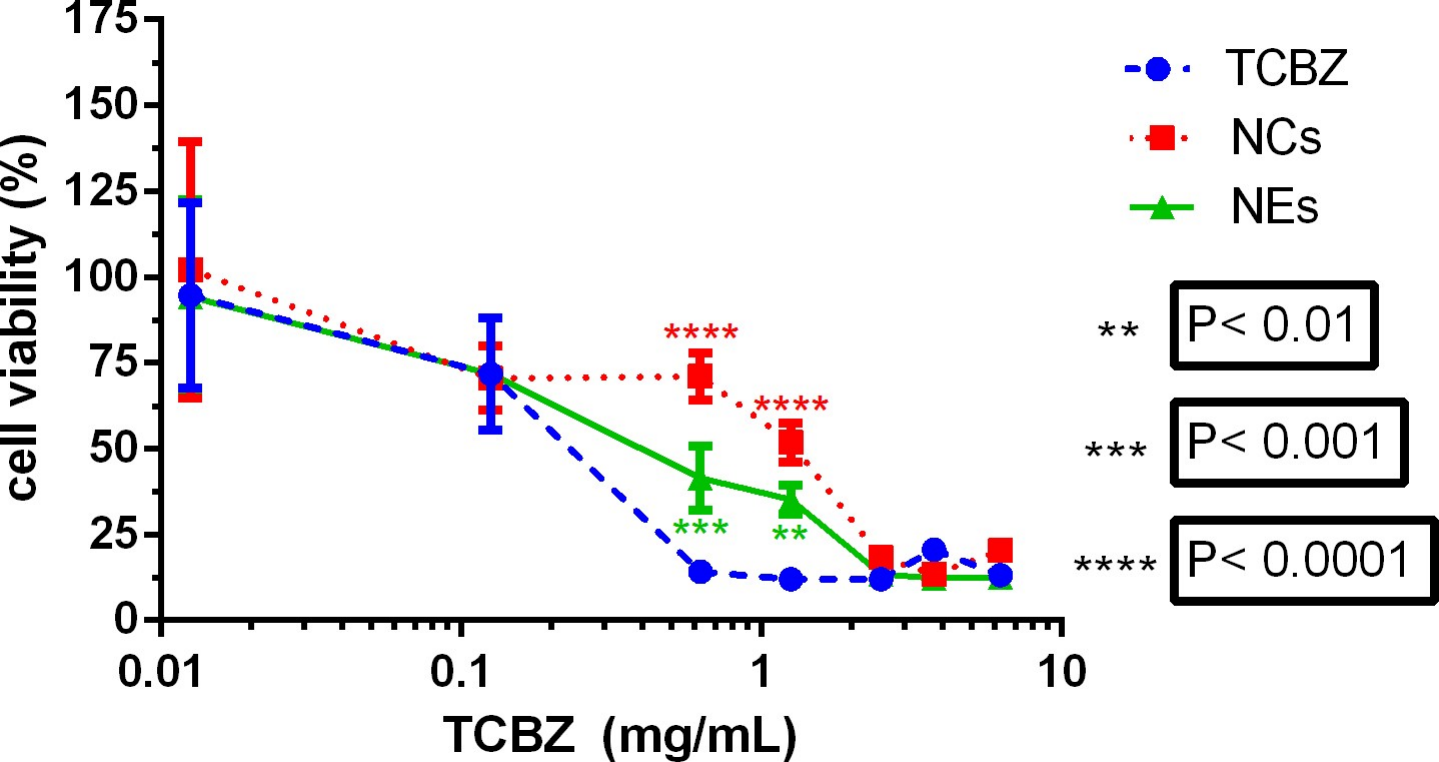
Cytotoxicity of raw and nanoencapsulated TCBZ (nanocapsules (NC), nanoemulsion (NE)) as determined by MTT assay on Caco-2 cells at varying TCBZ concentrations.

Fig 10 shows a comparison of cellular viability of Caco-2 cells treated with suspended TCBZ and TCBZ loaded in NEs and NCs over a range of concentrations. At concentrations lower than 0.1 mg/mL and higher than 2.5 mg/mL TCBZ no difference between dispersions, NCs and NEs was found. Cell viabilities between 75 and 100% were measured at concentrations lower than 0.1 mg/mL and cell viability lower than 10% at concentrations higher than 2.5 mg/mL for all treatments. Interestingly, at intermediate concentrations (0.625 mg/mL and 1.25 mg/mL TCBZ) both the NCs and NEs showed a lower cytotoxicity when compared to the dispersed drug. At 0.625 mg/mL TCBZ the NCs showed the lowest cytotoxicity at 71%, NEs 41%, whereas only 14% of cells that were treated with the drug dispersion were found viable.

The results of the cell viability study showed that at intermediary concentration, encapsulation of TCBZ can lower its cytotoxic effects when compared to the dispersed free drug. TCBZs cytotoxic effects are due to inhibition of nematode and mammalian α-tubuline [40]. A protective effect from drug cytotoxcity by nanoencapsulation of drug molecules has been shown previously for capsaicin in different cell lines *in vitro* [31,41]. Several studies have addressed the cytotoxicity of loaded and unloaded NEs and NCs in similar size ranges and found low cytotoxicity of unloaded formulations towards Caco-2 and MDCK cell lines thought to arise from the positive charge of the chitosan coating interfering with cell membrane components that carry a negative charge [34, 31]. In the case of TCBZ the low drug release we found in PBS at pH 7.4 is probably responsible for the decreased cytoxicity. The presence of cells and their lipophilic membranes as acceptors for lipophilic TCBZ might speed up the release of the drug significantly when compared with the release experiments in a strictly lipophobic environment, explaining the similarity in toxicitiy of raw and nanoencapsulated TCBZ at higher doses.

### Cellular localization studies

**Fig 11:**
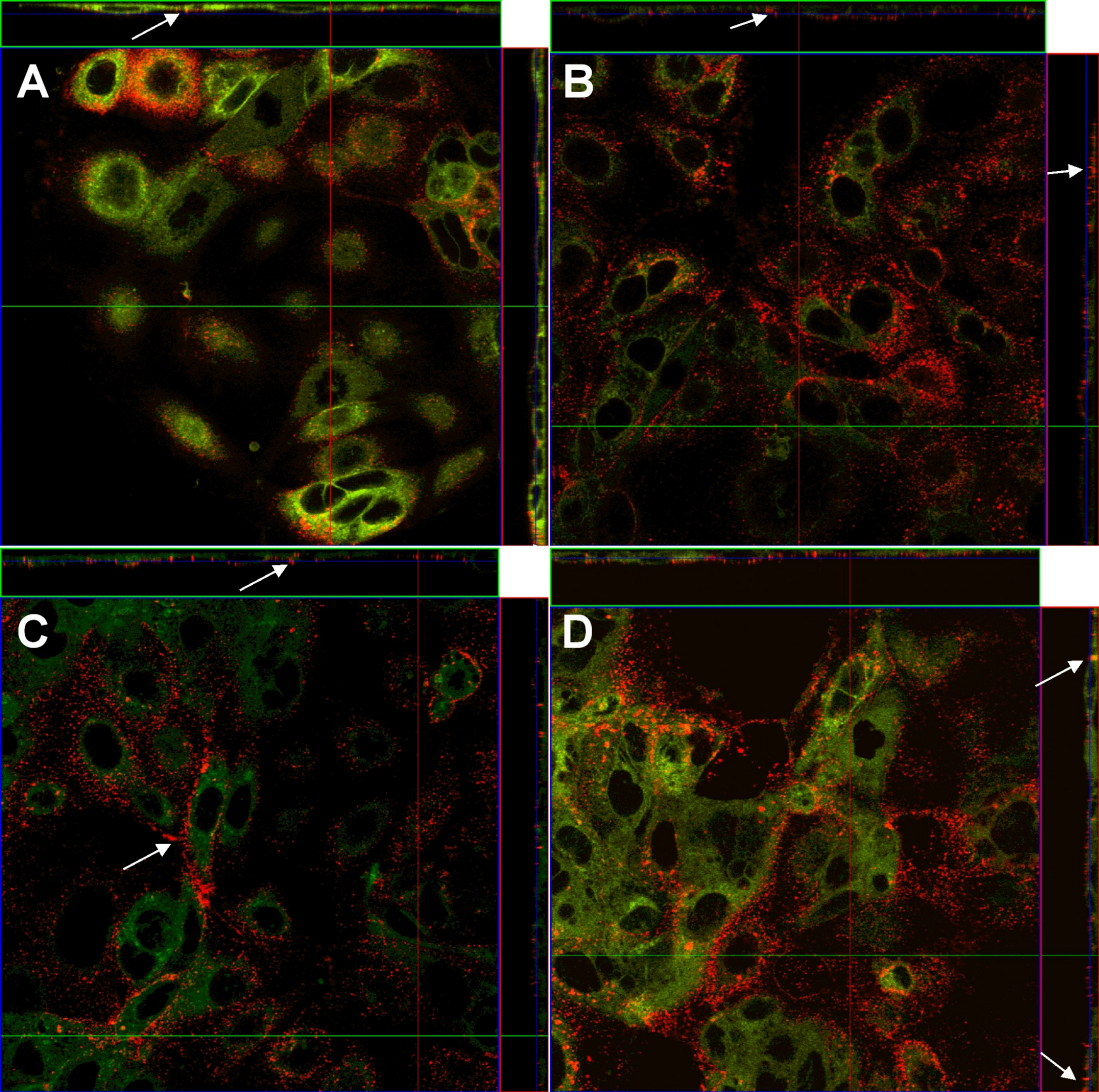
NCs accumulate on the cell surface and between Caco-2 cells. CLSM images of DiD labelled NCs in interaction with membrane stained Caco-2 cells at incubation times of 30 min (A), 60 min (B), 120 min (C) and 180 min (D). Images show top down view (main image) and orthogonal section indicated by the red line (right side of main image) and green line (top of main image). White arrows indicate aggregation of NCs in space between adjacent cells.

Fig 11 shows the interaction of fluorescent labelled NCs with Caco-2 cells. Due to insufficient stability in cell culture media, NEs could not be included in this microscopy studies. We found significant aggregation of NCs on the cell surfaces, especially in the space between adjacent cells, where NCs were able to penetrate the monolayer of cells. At 30 min a slightly lower number of NCs were found on the surface of cells, while all other incubation times showed similar patterns of surface aggregation and only minor amounts of cellular uptake.

The CLSM study has shown that CS-coated NCs strongly interacted with Caco-2 cells *in vitro* even after several washing steps and aggregated in the space between adjacent cells. Caco-2 cells possess tight junctions that can be remodelled due to nutrients present in the medium [42] and several studies have addressed the reversible opening of tight junctions due to presence of CS in several cell lines, including Caco-2 [43,44] which could explain the strong accumulation of CS-coated NCs. The strong interaction of NCs with mammalian cells could increase the passage time of TCBZ in oral drug delivery and thereby increasing its bioavailability, especially, when administered under fasting conditions [45]. Similar findings were made in a previous study with MDCK cells, where after 2 h some nanocapsules of similar size and surface charge interacted with the cell surface and only few were uptaken, which increased up to 24 h, although this is an unrealistic timespan for oral passage time of our formulation and the mechanism of cellular uptake is still unclear as of now [31].

## Conclusions

In this study, we have investigated the encapsulation of TCBZ into nanometer-sized emulsions and chitosan-coated capsules. To date, this is the first report on TCBZ nanoencapsulation. Higher loading of TCBZ increased the size of positively charged NCs while the size of negatively charged NEs was unaffected up to the point where both systems became unstable. Higher loading decreased loading efficiency, which was still higher than 50% at 18.75 mg/mL, compared to a predicted water solubility of TCBZ of 0.508 μg/mL. All formulations were stable in size over the time-course of one month at 4, 25 and 37°C but their zeta-potential was slightly decreased regardless of storage temperature. An increase of 9- to 16-fold was observed in the release of TCBZ from NCs and NEs, respectively, comparing with raw TCBZ in SGF, while almost no release was found in SIF. We correlated this higher release of TCBZ in SGF with the increased solubility at lower pH values due to possible ionization. Cytotoxicity studies with enterocytes revealed a cytoprotective effect of the NCs and NEs over drug dispersions between 0.1 and 2 mg/mL of TCBZ. Finally, we found that fluorescent labelled NCs strongly interact with enterocytes *in vitro* and thereby could enable a higher uptake and sustained release of TCBZ when compared to both NEs and drug dispersions. Thus, these findings exhibit relevant pharmaceutical potential in view of developing a novel TCBZ delivery oral formulations. Further studies should investigate whether higher solubility and adhesion of NCs translate into higher bioavailability in *in vivo* experiments and improve on currently available oral drug dispersion formulations.
